# Comprehensive Management of Pediatric Orbital Fractures: A Case Series and Review of Literature

**DOI:** 10.7759/cureus.57915

**Published:** 2024-04-09

**Authors:** Abiskar Basnet, Ashi Chug, Saurabh Simre, Akansha Vyas, Sudarshan Shrestha

**Affiliations:** 1 Oral and Maxillofacial Surgery, All India Institute of Medical Sciences, Rishikesh, Dehradun, IND; 2 Oral and Maxillofacial Surgery, All India Institute of Medical Sciences, Rishikesh, Rishikesh, IND; 3 Oral and Maxillofacial Surgery, All India Institute of Medical Sciences, Rishikesh, Uttarakhand, IND

**Keywords:** pediatric, orbital fracture, diplopia, muscles entrapment, oculocardiac reflex, trapdoor fracture

## Abstract

Orbital fractures in the pediatric population are rare. A trapdoor fracture is a special anatomic type of orbital fracture associated with herniation of orbital contents and entrapment of extraocular muscles entrapment with no signs of any soft tissue trauma. A delay in diagnosis can lead to a life-threatening condition known as oculocardiac reflex, characterized by nausea, vomiting, bradycardia, and syncope. Many authors recommend early surgical intervention, but some patients may delay for various reasons. We hereby represent three cases of orbital fracture to prevent long-term persistent diplopia. Depending on the case scenario, two cases were operated on in which an autogenous iliac cortical graft was placed in one patient to prevent postoperative herniation of orbital content, and in one patient, only release of entrapped muscles was done. One patient was managed conservatively with a regular follow-up visit.

## Introduction

Maxillofacial fractures are rarely evident in the pediatric population, with an incidence rate of less than 15% [1]. Among pediatric maxillofacial fractures, the orbital fracture is the least reported fracture type (9%) in comparison to mandibular (32.7%), nasal (30.2%), and maxillary/zygomatic fractures (28.6%) [2]. Soll and Poley were the first to identify the anatomic subtype of orbital blowout fracture known as trapdoor fracture [3]. Because of the greater elasticity of the facial bones, children and adolescents are more likely to experience it than adults [4]. Trapdoor fractures are pure orbital fractures, linear in form, and hinged medially, causing herniation of orbital contents into the maxillary sinus. The hinged fractured bone often snaps back to its original position, causing entrapment of the orbital contents. This entrapment of periorbital tissue results in severe oculocardiac reflex demonstrated by symptoms like nausea, vomiting, bradycardia, syncope [5], and other clinical manifestations like a limitation of extraocular muscle movement (EOM), diplopia, and painful, restricted eyeball movement [6].

White-eye blowout fracture was first introduced by Jordan et al. in 1998 [7]. Patients frequently present with symptomatic diplopia, severe restriction of eye movement, positive forced duction tests, entrapment of the orbital contents confirmed by computed tomography (CT) with symptoms of oculo-cardiac reflex, and in such cases, urgent surgery within 48 hours is recommended [8]. The decision of timely surgical intervention is critical to result in better clinical outcomes with surgical intervention. Management of blowout orbital fracture depends on the severity of symptoms. Criteria for orbital fractures can be categorized into three timeframes: immediate, early, and delayed. Persisting oculocardiac reflex symptoms such as nausea, vomiting, bradycardia, heart block, or syncope, along with signs of an entrapped muscle or periorbital tissue in the CT scan, requires immediate intervention within 24 hours also the condition where there is a notable displacement of the eyeball within the orbit, which poses a risk of serious vision-related complications requiring urgent medical attention. In the situation where double vision is experienced along with evidence of positive forced duction and a large floor fracture with less than 50% surface displacement seen in CT scan, leading to the development of latent enophthalmos requires early intervention within 1-14 days. Enophthalmos or hypoglobus presenting later on and symptomatic diplopia but no evidence of muscle entrapment on CT scan may require delayed intervention after 14 days [9]. If there are mild restrictions in eye movement (only soft tissue entrapment), a two-week observation is recommended. If symptomatic diplopia or restricted movements still persist after the edema has mostly resolved, surgical intervention is indicated [10]. Both surgical and conservative treatment have favorable outcomes, with a high proportion (96%) of surgically treated patients having no postoperative diplopia. Immediate release is more likely to be required for the age group of 0-12 years than for older patients [11]. In the present report, a compelling case series of three patients, shedding light on varied presentations and outcomes, is given by the authors.

## Case presentation

Case one

An 11-year-old male patient with an alleged history of sports injury reported to the emergency department with a prior history of four vomiting episodes in the past 24 hours. There was no reported record of loss of consciousness. At the time of presentation, the patient had hypotension and bradycardia. The patient had no medical comorbidities. The Glasgow Coma Scale of the patient was 15/15. Clinically, there were no visible facial fractures. On eye examination, there was no subconjunctival hemorrhage. The pupillary reaction was normal in both eyes. Extraocular muscle examination showed restriction of left eye movement in elevation, levo-elevation, dextroelevation, depression, dextro-depression, and levo-depression associated with binocular blurring of vision in levo-depression. No deformity or tenderness of the left orbital rim was detected.

**Figure 1:**
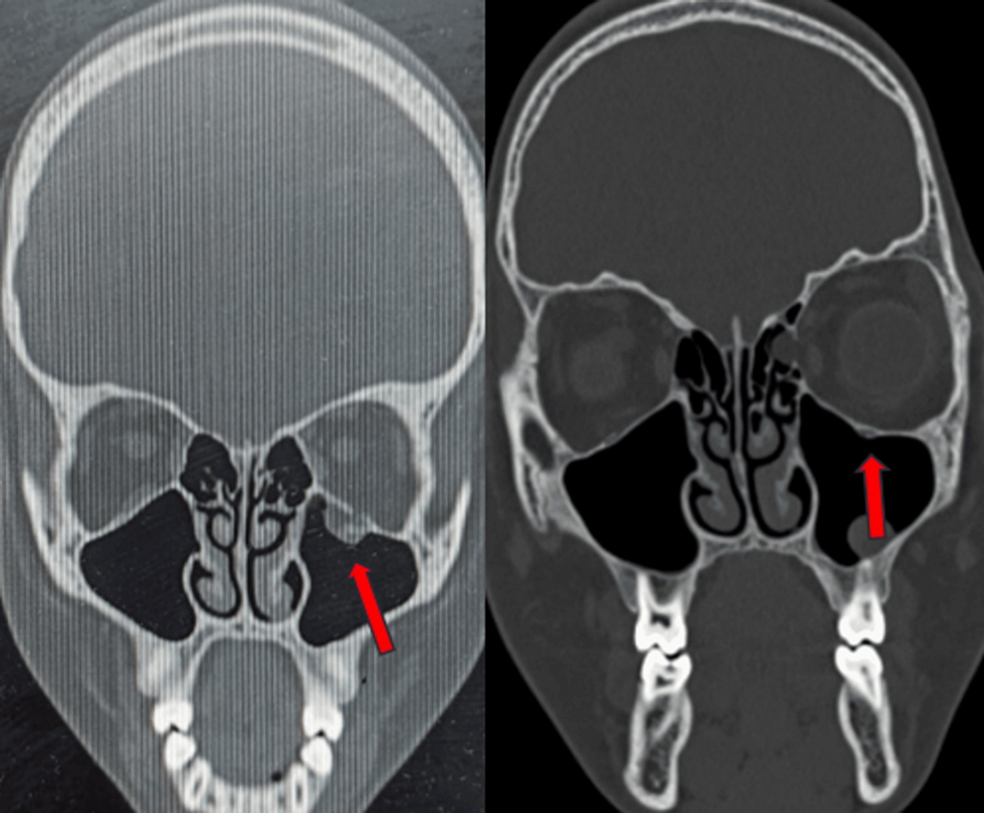
Case one: the coronal section of NCCT face shows pre and post, orbital floor fracture repair by iliac crest bone graft NCCT - non-contrast computed tomography

**Figure 2:**
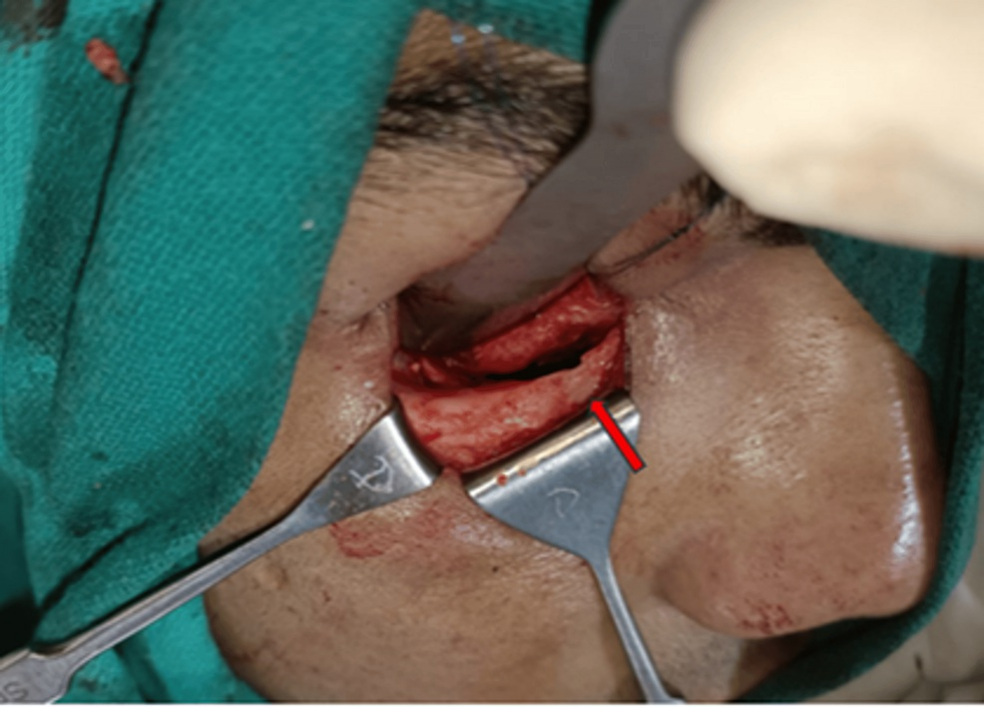
Intraoperative picture showing orbital floor fracture

Further CT examination of the orbit revealed a left inferior orbital floor fracture along with muscle entrapment and extraconal fat herniation into the left maxillary sinus. The patient was immediately intervened for surgical exploration of the left orbit via a transconjunctival approach. The orbital floor fracture, along with muscle entrapment, was identified. The entrapped muscle was carefully released, and the orbital floor defect was reconstructed using an autogenous cortical iliac graft. The intraoperative forced duction test revealed no restriction of eye movement. The range of movement improved significantly, along with the resolution of diplopia and pain in less than two weeks postoperatively. A Hess chart ascertained an unremarkable ocular motion (Figures 1, 2).

Case two

A seven-year-old male patient had an alleged history of a fall from a height of about 12 feet at his residence and was admitted for the management of a right neck femur fracture where he complained of double vision. An ophthalmology opinion was sought and he was diagnosed with a right orbital floor fracture along with muscle entrapment and referred to our department for management. There was no reported history of loss of consciousness, vomiting, or seizures post-injury. On eye examination, there was no subconjunctival hemorrhage. The pupillary reaction was normal in both eyes. Binocular diplopia was present with respect to dextro-depression and depression gazes. There was no blurring of vision nor restriction of eye movements to bilateral eyes.

**Figure 3:**
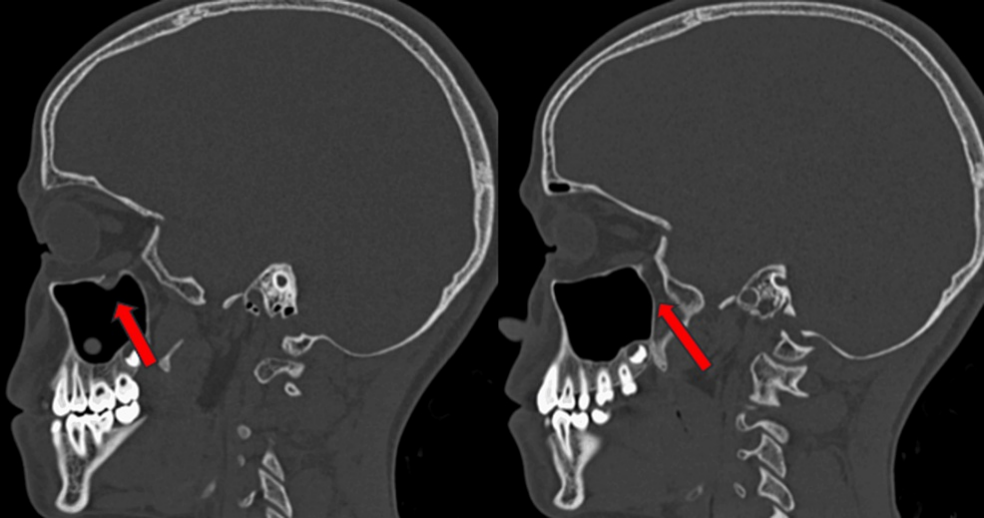
Case two: the sagittal section of NCCT face shows the release of entrapped muscles and orbital content and swing back to its position NCCT - non-contrast computed tomography

A fracture of the orbital floor with herniation of fat, along with entrapment of the inferior rectus into the right maxillary sinus, was reported in a CT scan. No obvious indentation was seen on the optic nerve. The patient underwent surgical exploration of the right eye through the subciliary approach, and herniated muscle and fat was released carefully. No restricted movement of the globe was found in all gazes on the forced duction test. Pain and diplopia resolved within one week postoperatively without restriction in eye movements (Figure 3).

Case three

A five-year-old boy presented to our department with an alleged history of a fall from a height of about 10 feet at his residence. There was no history of loss of consciousness, no vomiting, and no seizure post-injury. On inspection, the left eye had mild periorbital edema without subconjunctival hemorrhage or ecchymosis. The pupillary reaction was normal in both eyes. No restriction of eye movement was observed in bilateral eyes. Binocular diplopia was noted with depression gaze. On palpation, mild tenderness was present with respect to the left infraorbital rim, but no deformity was present.

**Figure 4:**
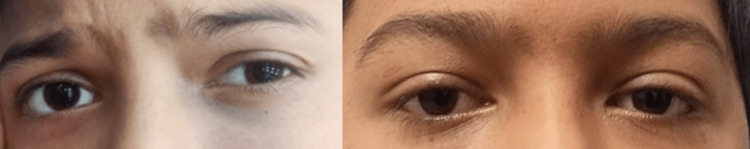
Case three: clinical picture reveals correction of diplopia after medications

A left orbital floor fracture with fat herniation into the left maxillary sinus was evident in the CT orbit. The optic nerve had no visible indentations. No immediate surgical action was taken. The patient was prescribed oral corticosteroid tabs of prednisolone 20mg once daily (OD) for the first two weeks, then tapered gradually with 10mg OD for the next week, 5mg OD for the next week, 5mg OD alternate day for a week, and then stopped and was monitored closely. After the follow-up period of two weeks, the discomfort and diplopia were completely resolved (Figure 4).

## Discussion

Trapdoor orbital fractures are more commonly seen in the pediatric population and usually result in the entrapment of orbital contents. Since there is no soft tissue injury, these fractures lead to diagnostic challenges. There is greater elasticity in the facial bones, which results in fractures with soft tissue and muscle entrapment. Broadly, the etiology of blowout fractures is attributed to three fundamental concepts: the hydraulic theory, the globe-to-wall contact theory, and the bone conduction theory [12]. The hydraulic theory was first put forward by King et al. in 1944 [13]. The force created by the sudden application of pressure by an object to the eyeball causes direct compression and fracture of the floor of the orbit.

The globe-to-wall contact theory advocated by Pfeiffer in 1943 described the globe as subjected to a force that propels it posteriorly into orbit, striking and breaking the bony orbital walls [14]. In the buckling mechanism, any force transmitted in an anteroposterior direction through the rim transiently shows a ripple effect and fractures the thin orbital floor.

Oculocardiac reflex is the clinical symptom often reported after a white-eye blowout fracture, which is a potentially life-threatening condition indicated by nausea, vomiting, headache, bradycardia, and hypotension leading to cardiac arrest. It is mediated by sensory afferents of the ophthalmic division of the trigeminal nerve combined with the visceral motor nucleus of the vagus nerve through the reticular formation in the brain stem [15].

A CT scan of the orbit is the desired imaging modality for the detection of suspected fractures of the orbital floor. In cases of acute trauma, the precise identification of herniated soft tissue at fracture sites may prove challenging. Hence, a crucial aspect lies in clinically assessing the entrapment of muscle or soft tissue along with the radiological correlation [16].

Prompt diagnosis and intervention of fractures of the orbit are crucial; otherwise, they might result in deformities, including globe malposition and, in severe cases, diplopia [12]. Early surgical intervention and release of entrapped muscle within 48 hours are recommended for superior outcomes. The rationale for early intervention is to avoid the likelihood of ischaemic necrosis, fibrosis, and scarring of entrapped muscles, which otherwise can progress to restricted ocular mobility and persistent diplopia [17]. The different surgical approaches that can be used for the exploration of the orbital floor. Herniated orbital contents should be dissected and released carefully. The defect of the orbital floor should be repaired adequately to prevent re-herniation and entrapment of periorbital fat/muscles postoperatively. An autogenous bone graft is preferred when a bony defect is larger than 50% of the floor of the orbit [18]. Autologous graft is considered the gold standard material for the repair of the floor of the orbit. The autologous graft is usually preferred for small-sized orbital floor defects (<2 cm^2^), while for large-sized defects, pre-bent titanium mesh or patient-specific implants are preferred [19]. Burnstine proposed criteria for the management of fractures of the floor of the orbit: immediate, within two weeks, and observation [20]. Early repair is done in those conditions in which muscle/soft tissue gets entrapped in the fracture line, causing serious functional restrictions. The mechanism is that the traumatizing force causes a rise in infraorbital pressure, which leads to a linear or trapdoor fracture of the floor or wall. Soft tissues around the orbit are propelled across the gap created by the fracture when it opens. Over time, avascular necrosis occurs, causing permanent damage to the muscle. Therefore, it warrants early recognition as a medical emergency and necessitates prompt intervention. Bradycardia, nausea, vomiting, syncope, and a non-resolving oculocardiac reflex are other reasons for urgent surgical intervention. Similarly, from the author's experience, it is necessary to undergo immediate release of muscle entrapment, as demonstrated in this report.

Patients with no eye movement restriction but presenting with characteristic diplopia and soft tissue entrapment in the line of fracture with little improvement over time can be treated within two weeks of injury. In these situations, waiting until the edema has resolved proves to be more effective in surgical repairs and minimizes the potential for globe damage. Similarly, in case three of this report, the patient reported persistent diplopia and entrapment of extraocular muscles on CT but no restriction of eye movement. As there was no bony defect, only the release of entrapped muscles was done. Regardless of the timing of surgery, patients experiencing muscle entrapment require a longer duration for recovery in the extraocular muscle movement and diplopia than entrapment of the soft tissue. Furthermore, there is a lower rate of extraocular muscle movement recovery in muscle entrapment patients in contrast to patients with involvement of soft tissue [21].

Observation and medical management can be done for orbital fractures associated with very mild diplopia and good extraocular movement, with a CT scan showing mild defects not supposed to cause enophthalmos and/or hypoglobus. Medical management, like antibiotics and steroids, is efficient in conservative management. In some cases of soft tissue entrapment with no evidence of muscle incarceration and minimal ocular motility restriction, conservative management can be done [5]. In one of our cases, there was entrapment of soft tissue evident in CT with no ocular motility restriction. We have thus proceeded with conservative management.

Because of direct injury to the extraocular muscle, persistent diplopia becomes a frequent side effect of trapdoor fractures in children. Orbital cellulitis is an uncommon sequela.

## Conclusions

Trapdoor fracture is predominantly seen in pediatrics and is the result of entrapment of extraocular muscle entrapment in the fractured area. A delay in diagnosis can yield potentially life-threatening conditions. Intervention within 48 hours provides optimum results. However, the decision is based on a thorough clinico-radiological evaluation and prompt diagnosis.
